# Supplementary material for: Bacterial flagella grow through an injection-diffusion mechanism
Source: eLife. 2017 Mar 6;6:e23136. doi: 10.7554/eLife.23136 (PMC5386592; doi:10.7554/eLife.23136)
Supplement: Figure 5—source data 1. — DOI: http://dx.doi.org/10.7554/eLife.23136.016 [file elife-23136-fig5-data1.doc]

**Figure 5-source data 1: Parameters *k_on_* and *D* of the injection-diffusion model fits of Figure 5 – Figure Supplement 3.**

| **Plasmid** | **∆t (min)** | ***k_on_* (s^-1^)** | ***D* (m^2^ ⋅ s^-1^)** |
| --- | --- | --- | --- |
| (-) | 30 | 28.24 | 5.51 × 10^-13^ |
| (-) | 60 | 28.22 | 5.51 × 10^-13^ |
| (-) | 90 | 28.26 | 5.51 × 10^-13^ |
| (-) | 120 | 27.33 | 5.32 × 10^-13^ |
| (-) | 150 | 26.99 | 5.25 × 10^-13^ |
| WT | 30 | 32.88 | 6.16 × 10^-13^ |
| WT | 60 | 33.42 | 6.27 × 10^-13^ |
| WT | 90 | 33.88 | 6.35 × 10^-13^ |
| WT | 120 | 32.85 | 6.15 × 10^-13^ |
| WT | 150 | 32.49 | 6.09 × 10^-13^ |
| ∆C_L_ | 30 | 29.62 | 5.80 × 10^-13^ |
| ∆C_L_ | 60 | 29.05 | 5.70 × 10^-13^ |
| ∆C_L_ | 90 | 29.02 | 5.69 × 10^-13^ |
| ∆C_L_ | 120 | 28.04 | 5.49 × 10^-13^ |
| ∆C_L_ | 150 | 27.90 | 5.46 × 10^-13^ |
| ∆N ∆C_L_ | 30 | 32.10 | 5.60 × 10^-13^ |
| ∆N ∆C_L_ | 60 | 32.04 | 5.59 × 10^-13^ |
| ∆N ∆C_L_ | 90 | 32.14 | 5.60 × 10^-13^ |
| ∆N ∆C_L_ | 120 | 31.31 | 5.46 × 10^-13^ |
| ∆N ∆C_L_ | 150 | 31.12 | 5.42 × 10^-13^ |
| ∆C_S_ | 30 | 33.57 | 5.91 × 10^-13^ |
| ∆C_S_ | 60 | 33.24 | 5.86 × 10^-13^ |
| ∆C_S_ | 90 | 32.98 | 5.80 × 10^-13^ |
| ∆C_S_ | 120 | 32.08 | 5.64 × 10^-13^ |
| ∆C_S_ | 150 | 31.88 | 5.60 × 10^-13^ |
| ∆N ∆C_S_ | 30 | 30.83 | 5.47 × 10^-13^ |
| ∆N ∆C_S_ | 60 | 30.98 | 5.50 × 10^-13^ |
| ∆N ∆C_S_ | 90 | 31.04 | 5.51 × 10^-13^ |
| ∆N ∆C_S_ | 120 | 30.21 | 5.36 × 10^-13^ |
| ∆N ∆C_S_ | 150 | 30.30 | 5.37 × 10^-13^ |
